# Innervated superficial circumflex iliac artery perforator flap for refractory elbow ulcer with bone exposure in Werner syndrome: A case report

**DOI:** 10.1016/j.jpra.2026.05.031

**Published:** 2026-05-20

**Authors:** Yuta Shimizu, Shoichi Imai, Kohei Mitsui, Megumi Furuya, Chihena Hansini Banda, Kanako Danno, Mitsunaga Narushima, Ryohei Ishiura

**Affiliations:** a Department of Plastic and Reconstructive Surgery, Kindai University Hospital, 1-14-1 Mihara-dai, Minami-ku, Sakai, Osaka 590-0197, Japan; b Department of Plastic and Reconstructive Surgery, Tokyo Metropolitan Police Hospital, 4-22-1 Nakano, Nakano-ku, Tokyo 164-8541, Japan; c Department of Plastic and Reconstructive Surgery, Mie University Hospital, 2-174 Edobashi, Tsu, Mie, Japan

**Keywords:** Werner syndrome, Superficial circumflex iliac artery perforator flap, Elbow ulcer, Bone exposure, Sensory reconstruction

## Abstract

Werner syndrome is a rare autosomal recessive progeroid disorder first described by Werner and is characterized by premature aging, progressive cutaneous and subcutaneous atrophy, impaired wound healing, and premature arteriosclerosis, which contribute to chronic refractory ulceration and may lead to bone exposure and functional impairment.

We report a case of a 52-year-old man with Werner syndrome who developed a refractory ulcer over the right olecranon with bone exposure. Reconstruction was performed using an innervated superficial circumflex iliac artery perforator (SCIP) flap, with vascular anastomosis to the inferior ulnar collateral artery and sensory neurorrhaphy to the medial antebrachial cutaneous nerve.

The postoperative course was uneventful. The flap survived completely, protective sensation was preserved, and elbow function improved. No ulcer recurrence or other complications were observed during the 5-year follow-up period.

This case suggests that an innervated SCIP flap can provide durable, sensate, and function-preserving reconstruction for refractory elbow ulcer with bone exposure in Werner syndrome.

## Introduction

Werner syndrome is a rare autosomal recessive progeroid disorder.^1^ Clinically, affected patients often present with characteristic features such as short stature**,** premature graying, bilateral cataracts, diabetes mellitus, cutaneous atrophy, and an increased risk of malignancy.^2^ Its pathophysiological features include progressive cutaneous and subcutaneous atrophy, impaired wound healing, and premature arteriosclerosis.^3^ These changes predispose patients to chronic refractory ulceration, particularly in the extremities, where ulcers may progress to deep soft-tissue loss or bone exposure.^4^

Reconstruction of ulcers in Werner syndrome is particularly challenging because both local tissue quality and systemic vascular status may be compromised.^3^ Skin grafts and local flaps may be unreliable because of dermal fragility, subcutaneous tissue atrophy, and poor vascularity. In addition, premature arteriosclerosis and other comorbidities may further complicate free tissue transfer. Therefore, reconstruction requires not only durable soft-tissue coverage but also careful consideration of flap selection, recipient vessels, and long-term resistance to recurrent breakdown.^4^

The SCIP flap provides thin and pliable vascularized tissue with minimal donor-site morbidity and has been used in extremity reconstruction.^5^ These characteristics may be advantageous for reconstruction of the elbow, where preservation of contour and joint mobility is important. In addition, sensory reconstruction may be beneficial in patients at risk of repeated tissue injury, although its role in Werner syndrome has rarely been discussed.

Here, we present a case of refractory elbow ulcer with bone exposure in a patient with Werner syndrome successfully reconstructed using an innervated SCIP flap. This case highlights a reconstructive strategy for achieving durable coverage, preservation of elbow function, and recovery of protective sensation in a condition associated with impaired wound healing and recurrent ulceration.

## Case presentation

### Patient information

A 52-year-old man presented with a refractory ulcer on the right elbow. He had no history of trauma or deep vein thrombosis. Physical examination showed short stature (153 cm, 33.0 kg), slender extremities, premature graying, a beak-shaped nose, a high-pitched voice, and bilateral juvenile cataracts. Multiple chronic ulcers with depressed scars and hyperpigmentation were noted, with the most severe lesion on the right elbow showing olecranon exposure. Radiography demonstrated Achilles tendon calcification. Based on these characteristic findings, Werner syndrome was diagnosed clinically.

### Physical examination

**Fig. 1:**
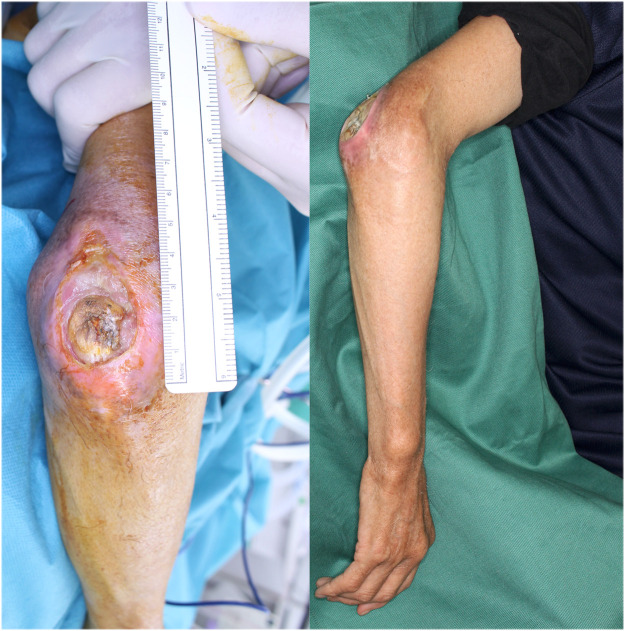
Preoperative photograph of the right elbow ulcer showing exposed olecranon.

The right elbow ulcer measured 2.5 × 4.0 cm, with bone exposure and surrounding sclerotic skin (Fig. 1). Elbow range of motion was 80° in flexion with a 50° extension deficit. Signs of infection were observed at the ulcer base.

### Investigations

Preoperative ultrasonography and CT angiography identified the inferior ulnar collateral artery as a suitable recipient vessel, with adequate patency and caliber for microvascular anastomosis. In the right groin, imaging demonstrated a superficial branch arising from the superficial circumflex iliac artery (SCIAs), which was marked preoperatively and selected as the pedicle for flap elevation.

### Surgical procedure

**Fig. 2:**
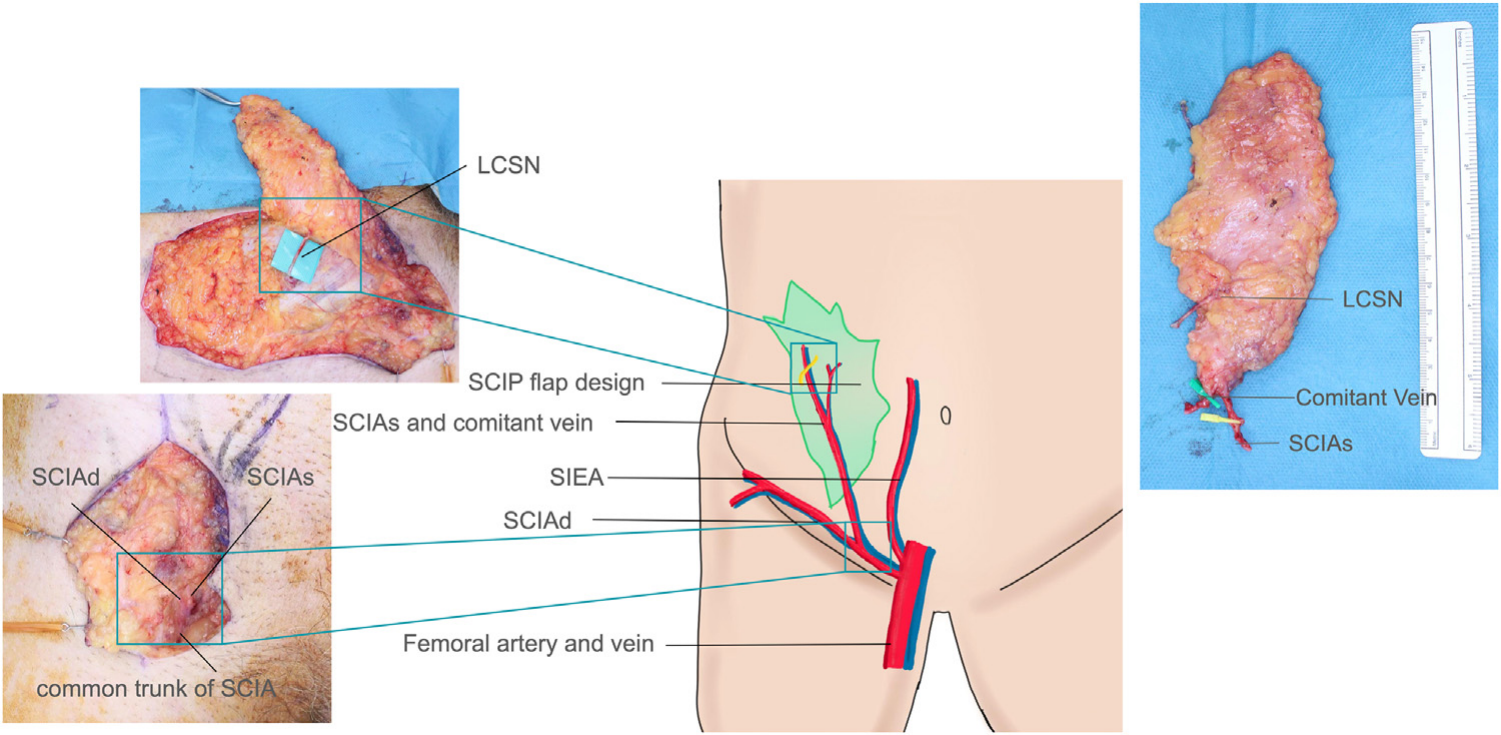
A 12 × 7 cm SCIP flap was harvested from the right groin. Schematic illustration and intraoperative pictures of flap pedicle, topographical vascular anatomy, including SCIAd, SCIAs, SIEA, the femoral artery, and LCSN. SCIAs and SCIAd had a common trunk. Harvested SCIP flap containing the SCIAs, its comitant vein, and the LCSN.

**Fig. 3:**
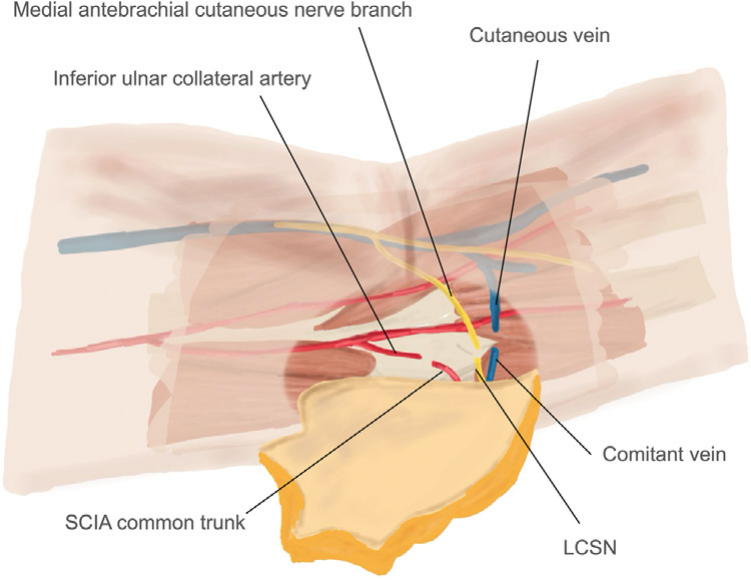
Schematic illustrations of SCIP flap transfer, showing flap inset: the SCIA common trunk was anastomosed to the inferior ulnar collateral artery, the comitant vein to a superficial vein, and sensory reconstruction was achieved by neurorrhaphy between LCSN and medial antebrachial cutaneous nerve branch.

A 12 × 7 cm SCIP flap was designed in the right inguinal region. After debridement of the ulcer, the flap was elevated from proximal to distal beneath the superficial fascia, incorporating a branch of the lateral cutaneous branch of the subcostal nerve (LCSN). The deep branch of SCIA (SCIAd) was ligated, and the pedicle was based on the superficial branch (Fig. 2). At the recipient site, the exposed olecranon was debrided. The inferior ulnar collateral artery (1.5 mm) was prepared as the recipient artery. End-to-end arterial anastomosis was performed between the SCIA common trunk (2.0 mm) and the inferior ulnar collateral artery. Venous anastomosis was performed between the SCIA comitant vein (2.0 mm) and a cutaneous vein (1.5 mm). Neurorrhaphy was performed between the LCSN and the medial antebrachial cutaneous nerve (Fig. 3). The distal portion of the flap was inset into the circular defect to interdigitate with the surrounding poorly vascularized tissue. Artificial dermis was applied over the anastomosis site to avoid compression of the vascular pedicle associated with direct closure. The donor site was closed primarily.

### Postoperative course and follow-up

**Fig. 4:**
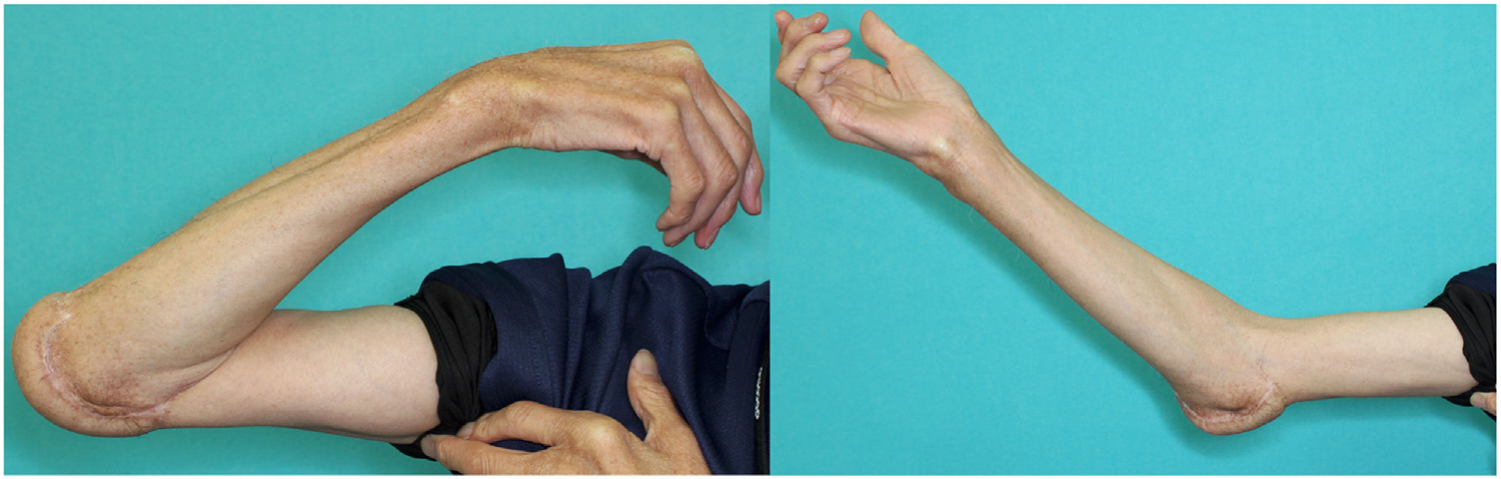
Functional outcomes at 5 years postoperatively. Elbow range of motion improved from 80° in flexion and 50° in extension preoperatively to 150° in flexion and 45° in extension postoperatively. The patient regained independence in daily activities, including wheelchair operation, brushing his teeth, and washing his hair.

The postoperative course was uneventful. The area covered with artificial dermis epithelialized uneventfully within 4 weeks with topical ointment treatment alone, without the need for additional surgical intervention. Prostaglandin E₁ (40 µg) was administered intravenously twice daily for 7 days. Protective sensation became clinically detectable from 1 year postoperatively. The flap remained viable, with preserved protective sensation and improved elbow function. At the 5-year follow-up, the Semmes–Weinstein monofilament test showed 3.22 (0.16 g) on the contralateral side and 4.56 (4.0 g) on the reconstructed side. Elbow range of motion improved from 80° in flexion with a 50° extension deficit preoperatively to 150° in flexion with a 45° extension deficit postoperatively (Fig. 4). The patient regained independence in daily activities, including wheelchair operation, brushing his teeth, and washing his hair. No ulcer recurrence, donor-site morbidity, or other complications were observed during the 5-year follow-up period.

## Discussion

Werner syndrome is caused by biallelic mutations in the WRN gene and is characterized by progressive cutaneous and subcutaneous atrophy, impaired wound healing, and premature arteriosclerosis.^6^ These pathophysiological features predispose affected patients to chronic refractory ulceration, particularly in the extremities, and may ultimately result in deep soft-tissue loss or bone exposure.^3^^,^^7^ Reconstruction in such patients is challenging because both local tissue quality and vascular status may be compromised.

Conventional reconstructive options are often unreliable in Werner syndrome. Skin grafting may fail because of poor vascularity, subcutaneous tissue atrophy, and insufficient wound bed quality.^8^ Local flaps may also be limited because adjacent tissues can be affected by similar degenerative changes. In such settings, free tissue transfer may offer more durable coverage. However, free flap reconstruction in Werner syndrome also requires careful preoperative planning because systemic vascular pathology and associated comorbidities may affect recipient vessel quality and flap safety.^3^ In the present case, preoperative ultrasonography and CT angiography confirmed the patency and adequate caliber of the brachial artery and inferior ulnar collateral artery, allowing safe microvascular reconstruction.

The elbow presents an additional reconstructive challenge because it is a mobile region with limited soft-tissue coverage over a bony prominence. For such a site, the reconstructive goal is not only durable coverage of the exposed bone but also preservation of joint mobility and contour. The SCIP flap was selected in this case because it provides thin and pliable vascularized tissue with minimal donor-site morbidity.^5^ In addition, previous studies have shown that superthin SCIP flaps can be harvested reliably for reconstruction requiring thin and flexible coverage.^9^ Although a bulkier flap may theoretically provide greater padding, excessive tissue volume may impair contour and joint mobility at the elbow. In the present patient, this low-bulk flap achieved stable coverage of the olecranon without restricting elbow motion, and no recurrence was observed during the 5-year follow-up period. These findings suggest that, in selected patients with Werner syndrome, a thin free flap can still provide sufficient long-term durability when appropriately vascularized tissue is transferred.

Alternative perforator flap options for upper-extremity reconstruction include the anterolateral thigh flap, medial arm perforator flap, and thoracodorsal artery perforator flap. However, these options were considered less suitable in the present case. A bulkier flap may impair contour and joint mobility at the elbow, which is a mobile and contour-sensitive region. In addition, tissue affected by similar degenerative changes associated with Werner syndrome was preferably avoided. Forearm-based flaps were also considered less favorable because they may transfer tissue with characteristics similar to those of the recipient site. In contrast, the SCIP flap provided thin and pliable vascularized tissue from a donor site less likely to be affected by chronic ulceration.

Sensory reconstruction in a patient with a WRN gene mutation has been reported previously, with partial sensory recovery after 6 months and protective sensation achieved at 12 months.^10^ In the present case, neurorrhaphy was performed between the lateral cutaneous branch of the subcostal nerve and the medial antebrachial cutaneous nerve, and protective sensation became clinically detectable from 1 year postoperatively. This postoperative course was similar to that of the previous report. The previous report also noted that protective sensation allowed the patient to wear shoes and walk normally. In the present case, elbow function was preserved and the patient regained independence in activities of daily living, including wheelchair operation, brushing his teeth, and washing his hair. Although the contribution of sensory reconstruction cannot be determined from a single case, postoperative recovery of protective sensation was confirmed. The contralateral side was used as an internal reference; however, some influence of the underlying disease on baseline sensation cannot be excluded.

Another advantage of the SCIP flap in Werner syndrome is donor-site selection. Ulcers associated with Werner syndrome predominantly affect the extremities, whereas trunk involvement is less common.^2^^,^^4^ Harvesting tissue from the groin therefore avoids areas that are more likely to be affected by chronic ulceration and, in the present case, enabled primary closure with minimal donor-site burden.

This report is limited by its single-case nature, and the superiority of an innervated SCIP flap over other reconstructive options cannot be determined. Nevertheless, this case demonstrates that an innervated SCIP flap can provide durable, sensate, and function-preserving reconstruction for refractory elbow ulcer with bone exposure in Werner syndrome. This approach may represent a useful reconstructive option when thin but stable coverage and preservation of joint mobility are both required.

## Ethical approval

Not required.

## Data availability statement

All data generated or analyzed during this case report are included in this published article.

## Funding statement

This case report did not receive any specific grant from funding agencies in the public, commercial, or not-for-profit sectors.

## Authors’ contributions

Y.S. conceived the case, collected data, and drafted the manuscript. S.I., K.M., M.F., C.H.B., K.D., and M.N. contributed to data interpretation and manuscript review. R.I. conceptualization, study design, data collection, surgical procedures, and manuscript drafting. All authors read and approved the final manuscript.

## Declaration of competing interest

The authors declare that they have no conflict of interest.
